# The N-glycome of human embryonic stem cells

**DOI:** 10.1186/1471-2121-10-42

**Published:** 2009-06-02

**Authors:** Tero Satomaa, Annamari Heiskanen, Milla Mikkola, Cia Olsson, Maria Blomqvist, Minna Tiittanen, Taina Jaatinen, Olli Aitio, Anne Olonen, Jari Helin, Jukka Hiltunen, Jari Natunen, Timo Tuuri, Timo Otonkoski, Juhani Saarinen, Jarmo Laine

**Affiliations:** 1 Glykos Finland Ltd, Helsinki, Finland; 2 Biomedicum Stem Cell Center, University of Helsinki, Helsinki, Finland; 3 Family Federation of Finland, Infertility Clinic, Helsinki, Finland; 4 Finnish Red Cross Blood Service, Helsinki, Finland; 5 Program in Structural Biology and Biophysics, Institute of Biotechnology/NMR Laboratory, University of Helsinki, Helsinki, Finland; 6 Hospital for Children and Adolescents, Helsinki University Central Hospital, Helsinki, Finland; 7 Current address: Nexstim Ltd., Helsinki, Finland

## Abstract

**Background:**

Complex carbohydrate structures, glycans, are essential components of glycoproteins, glycolipids, and proteoglycans. While individual glycan structures including the SSEA and Tra antigens are already used to define undifferentiated human embryonic stem cells (hESC), the whole spectrum of stem cell glycans has remained unknown. We undertook a global study of the asparagine-linked glycoprotein glycans (N-glycans) of hESC and their differentiated progeny using MALDI-TOF mass spectrometric and NMR spectroscopic profiling. Structural analyses were performed by specific glycosidase enzymes and mass spectrometric fragmentation analyses.

**Results:**

The data demonstrated that hESC have a characteristic N-glycome which consists of both a constant part and a variable part that changes during hESC differentiation. hESC-associated N-glycans were downregulated and new structures emerged in the differentiated cells. Previously mouse embryonic stem cells have been associated with complex fucosylation by use of SSEA-1 antibody. In the present study we found that complex fucosylation was the most characteristic glycosylation feature also in undifferentiated hESC. The most abundant complex fucosylated structures were Le^x ^and H type 2 antennae in sialylated complex-type N-glycans.

**Conclusion:**

The N-glycan phenotype of hESC was shown to reflect their differentiation stage. During differentiation, hESC-associated N-glycan features were replaced by differentiated cell-associated structures. The results indicated that hESC differentiation stage can be determined by direct analysis of the N-glycan profile. These results provide the first overview of the N-glycan profile of hESC and form the basis for future strategies to target stem cell glycans.

## Background

During the last decade global genomics and proteomics analyses of defined cell populations have revolutionized our understanding of cell biology. Glycomics – the study of global glycan expression profiles – has been predicted to be a next step forward [1]. Glycans, the carbohydrate units of glycoproteins, glycolipids, and proteoglycans, are capable of great structural variation and their specific molecular structures carry vast amounts of biological information [2]. It has been estimated that more than half of all cellular proteins are glycosylated [3], but little is known of glycan structures in specific cell types. Glycans linked to cell surface proteins and lipids form a dense layer – the glycocalyx – on the extracellular side of the cell surface. The glycocalyx has first-line functions in the communication of the cell and its environment, including both cell-to-cell contacts [2,4-6] and interactions with extracellular matrix components [7]. In addition, the specific roles of N-glycans involve regulation and control of protein folding [8,9] and trafficking [10].

Human embryonic stem cells (hESC) [11] provide models for the study of human development and toxicology and have therapeutic potential in regenerative medicine [12]. To effectively utilize these cells, novel differentiation stage and lineage specific stem cell markers are required. Since glycans are abundant components of the cell surface, reagents that specifically recognize hESC glycans should be useful tools for the identification, isolation, and manipulation of stem cells. In fact, the monoclonal antibodies currently used to define hESC, including the globo-series glycosphingolipid epitopes SSEA-3 and SSEA-4, and the keratanase-sensitive glycoprotein associated epitopes Tra 1–60 and Tra 1–81, recognize glycan antigens [13-15]. Further, the expansion of undifferentiated hESC and the directed differentiation of hESC to specific progeny lineages in cell culture remain problematic. Understanding how cells interact through the glycocalyx with feeder cells and other components of the culture environment may enable rational design of specific culture systems.

In the present study, a global analysis of the asparagine-linked glycans (N-glycans) of hESC and cells differentiated from them was performed by mass spectrometric profiling of unmodified glycans. We found that hESC have a characteristic and complex protein N-glycosylation profile. The data provide insight into the glycobiology of hESC and can be utilized as a basis for future studies exploring the role of stem cell glycans.

## Results

### Analysis strategy

**Figure 1:**
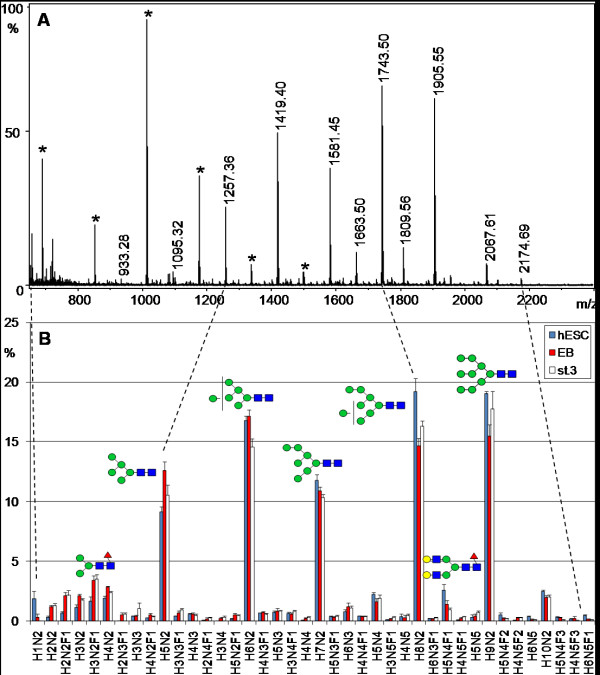
**Mass spectrometric neutral N-glycan profile of human embryonic stem cells (hESC)**. **A**. MALDI-TOF MS spectrum of neutral N-glycan fraction isolated from a hESC sample. **B**. Average of relative signal intensities from 40 most abundant neutral N-glycans of four finnish hESC lines (blue columns), embryoid bodies derived from the hESC lines (EB, red columns), and stage 3 differentiated cells (st.3, white columns). The columns indicate the mean abundance of each glycan signal (% of the total detected glycan signals). Error bars represent the standard error of mean. Proposed monosaccharide compositions are indicated on the x-axis and proposed structures for the major N-glycans are shown as schematic drawings. Gray circle/H: hexose, green circle: mannose, yellow circle: galactose, blue circle: glucose, gray square/N: N-acetylhexosamine, blue square: N-acetylglucosamine, red triangle/F: fucose/deoxyhexose, violet diamond/S: N-acetylneuraminic acid, light blue diamond/G: N-glycolylneuraminic acid. Asterisks indicate known polyhexose contamination that was not included in panel B.

**Figure 2:**
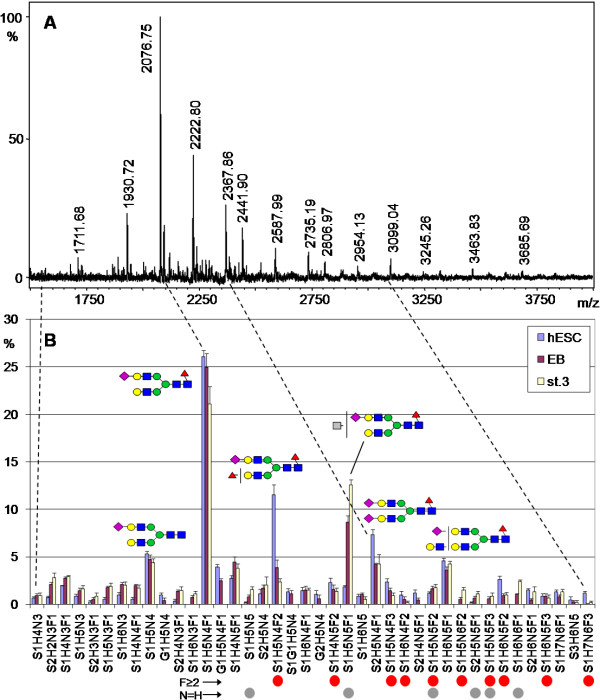
**Mass spectrometric acidic N-glycan profile of hESC**. **A**. MALDI-TOF MS spectrum of acidic N-glycan fraction isolated from a hESC sample. **B**. Average of relative signal intensities from 40 most abundant sialylated N-glycans of the four hESC lines, EBs, and stage 3 differentiated cells. N-glycan signals carrying structural features associated with either hESC (F ≥ 2, red circles) or differentiated cells (N = H, gray circles) are indicated below panel B. See the legend of Figure 1 for details and monosaccharide symbols.

In order to generate mass spectrometric glycan profiles of hESC, embryoid bodies (EB), and further differentiated cells, a matrix-assisted laser desorption-ionization (MALDI-TOF) mass spectrometry based analysis was performed. We focused on the most common type of protein post-translational modifications, N-glycans, which were enzymatically released from cellular glycoproteins. During glycan isolation and purification, the total N-glycan pool was separated by an ion-exchange step into neutral N-glycans and sialylated N-glycans. These two glycan fractions were then analyzed separately by mass spectrometric profiling (Fig. 1 and 2), which yielded a global view of the N-glycan repertoire and allowed comparative analysis of differentiation-associated changes. The present scarce sample amounts did not allow us to purify individual glycan components for structural analyses. However, detailed structural analyses were achieved from the total neutral and acidic N-glycan pools by a combination of proton NMR spectroscopy, specific glycosidase digestions, and MS/MS fragmentation experiments.

MALDI-TOF mass spectrometric analysis has been shown to be accurate in relative quantitation of glycan components within complex glycan mixtures [16]. We and others have found that it is a powerful tool for comparing changes in the glycan composition especially between closely related samples [17-19]. The present MALDI-TOF mass spectrometric methods have been optimized for relative quantitation of non-derivatized glycans and potentially labile glycan residues such as sialic acids are reliably determined. This was further verified during the present work by comparison of non-derivatized and permethylated glycan samples. The relative proportions of the major glycan signals were similar and no sialic acid or fucose loss was detected (data not shown). Due to the large m/z range of observed glycan ions the percentage of each glycan signal might not represent absolute molar percentage of the total cellular N-glycome. The amounts of especially larger glycans may be underestimated. However, we demonstrated with standard molecules that over the m/z range there was a linear response between the amount of added analyte and its relative signal intensity in the recorded glycan profile (see Additional File 1, Supplementary Fig. 1).

Each step in the glycan purification sequence was controlled for reproducibility by mixtures of glycoproteins, synthetic glycans, and glycan mixtures extracted from human cells. We routinely obtained the same mass spectrometric profiles for the standard glycan mixtures after each purification step during the multi-stage purification process [18] showing that there was no selective loss of glycans during purification. The yield of the N-glycosidase reaction was over 95% with model glycoproteins. The robustness of the present method was further evaluated by subjecting human cell samples to blinded analysis by five different persons. The results were highly comparable showing that the present method reliably reproduced the characteristic glycan profile of each cell type (see Additional File 1, Supplementary Fig. 2). The glycan profile was demonstrated to be reproducible also when different dilutions of the same sample were analyzed, demonstrating that the results were not sensitive to the exact amount of cells in each sample. However, in the present analyses the cell amounts were comparable in the whole sample series.

We analyzed N-glycan profiles of all the biological materials that were in contact with the stem cells and could potentially contaminate the samples with glycoproteins, including hEF and mEF feeder cells, cell culture media, and culture media supplements. The N-glycan profiles revealed that the employed cell harvesting and washing procedures had been efficient and neither the feeder cells nor the culture media glycoproteins had affected the observed stem cell N-glycan profiles to a marked extent. By comparing the characteristic glycan signals in each potential contamination source, we could calculate that N-glycan purity in the samples was at least 93%.

### hESC N-glycan profiles

Neutral N-glycans comprised approximately two thirds of the combined neutral and sialylated N-glycan pools of hESC. The relative proportions of the two glycan pools were analyzed by combining corresponding aliquots from the neutral glycan pool and the acidic glycan pool after broad-range sialidase digestion and comparing the combined glycan profile to the separate neutral and acidic glycan profiles (data not shown).

The 40 most abundant neutral N-glycan signals detected in the four hESC lines are presented in Figure 1B (blue columns). The similarity of the profiles with the four hESC lines, which is indicated by the minor variation in the glycan signals, suggests that the four cell lines closely resembled each other. For example, 15 of the 20 most abundant glycan signals were the same in every hESC line. The five most abundant signals (H5N2, H6N2, H7N2, H8N2, and H9N2) comprised 76% of the neutral N-glycan signals and dominated the profile.

All the major N-glycan signals in the acidic N-glycan fraction (Fig. 2B, blue columns) contained sialic acid residues. There was more variation between individual cell lines in the 40 most abundant acidic N-glycans than in the neutral N-glycans. However, the four hESC lines again resembled each other and the five most abundant sialylated N-glycan signals were the same in every cell line: S1H5N4F1, S1H5N4F2, S2H5N4F1, S1H5N4, and S1H6N5F1. The most abundant sialylated glycan signals contained the H5N4 core composition and differed only by variable number of sialic acid (S or G) and deoxyhexose (F) residues. These biantennary-size N-glycans together comprised 61% of the total glycan signal intensity in the acidic glycan fraction.

We detected N-glycans containing N-glycolylneuraminic acid (G) in the hESC samples, for example glycans G1H5N4, G1S1H5N4, and G2H5N4. N-glycolylneuraminic acid has previously been reported in hESC as an antigen transferred from culture media containing animal-derived materials [20]. Accordingly, the serum replacement medium used in the present experiments contained bovine serum glycoproteins. We have recently detected Neu5Gc in N-glycans of hESC and *in vitro *cultured human mesenchymal stem cells by mass spectrometric N-glycan analysis [21].

The four hESC lines shared the same overall N-glycan profile and there was only slight cell line specific variation within the profiles. The 30 most common N-glycan signals were the same in all the hESC lines and accounted for circa 85% of the total detected N-glycans. Similarly, EBs derived from each hESC line produced N-glycan patterns with similar characteristics, regardless of the different starting hESC line.

### Changes in the N-glycan profile during hESC differentiation

A major goal of the present study was to identify glycan structures that would be specific to either stem cells or differentiated cells, and could therefore serve as differentiation stage markers. In order to determine whether the hESC N-glycome undergoes changes during differentiation, the N-glycan profiles obtained from hESC, EB, and stage 3 differentiated cells were compared (Fig. 1B and 2B). The profiles of the differentiated cell types (EB and stage 3 differentiated cells) were clearly different compared to the profiles of undifferentiated hESC, as indicated by non-overlapping distribution bars in many glycan signals. This suggested that differentiation induced the appearance of new N-glycan types while earlier glycan types disappeared.

**Figure 3:**
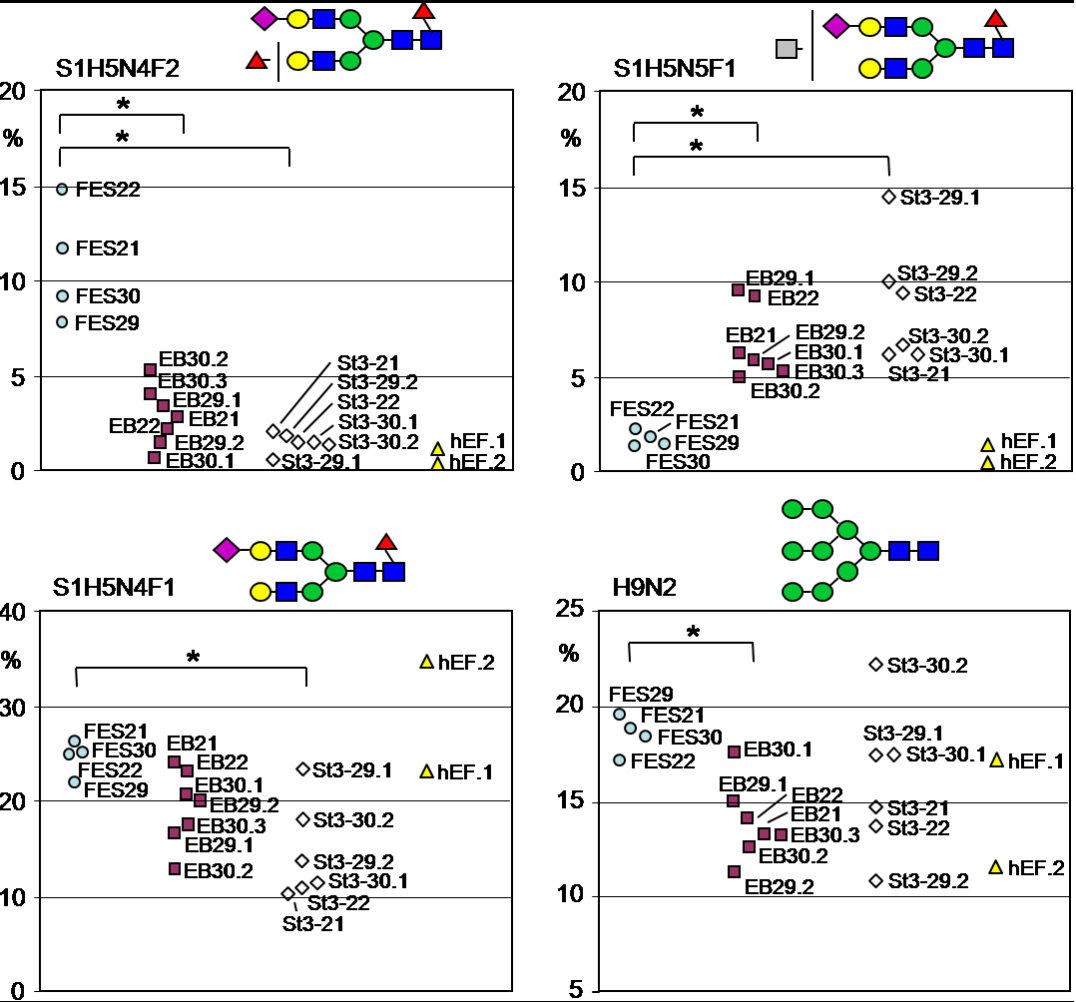
**Sample-to-sample comparison of relative signal intensities of four major N-glycan signals**. Sample-to-sample comparison of relative signal intensities of four major N-glycan signals in the whole dataset of four hESC lines (FES 21, FES 22, FES 29, and FES 30), embryoid bodies derived from them (EB), stage 3 differentiated cells (st.3), and two human fibroblast samples from the same cell line that was used as feeder cells in the propagation of hESC. **A**. Glycan signal S1H5N4F2 is characteristic to hESC. **B**. Glycan signal S1H5N5F1 is characteristic to differentiated cells. **C. and D**. The major N-glycan signals S1H5N4F1 (**C**.) and H9N2 (**D**.) are abundantly expressed in hESC and show variable expression in the differentiated cell types. *Asterisks *mark statistical significant differences between sample groups according to one-way ANOVA (p < 0.05).

Figure 3 presents the observed sample-to-sample variation in relative abundance of four N-glycan signals: S1H5N4F2, S1H5N4F1, and H9N2 (hESC-associated), as well as S1H5N5F1 (differentiated cell-associated). S1H5N4F2 (Fig. 3A) was the major hESC-specific glycan signal and had high abundance in all the four hESC lines, while its relative amount dropped consistently in all samples from both EB and further differentiated cells. In contrast, S1H5N5F1 (Fig. 3B) that was the major differentiation-associated glycan signal, was highly abundant in all EB and further differentiated cell samples, while it was only a minor glycan in all hESC samples. These two glycan signals could serve as markers of either the stem cells or the differentiated cells, respectively. The relative signal intensities of both S1H5N4F1 (Fig. 3C) and H9N2 (Fig. 3D) were on average slightly higher in hESC than in the differentiated cells, but individual differentiated cell samples differed greatly from the average. These glycan signals may therefore not be useful differentiation stage markers as such, but may however indicate trends in glycan biosynthesis.

The major differentiation stage associated N-glycan signal types were also visualized in the sialylated N-glycan profiles (Fig. 2B). There was a significant hESC association in the glycans S1H5N4F2 (Fig. 3A), S1H6N5F2, and S1H5N4F3 as well as other glycan signals that contained at least two deoxyhexose residues (F ≥ 2; marked with red circles under Fig. 2B). Even minor signals with this structural feature were more abundant in hESC than in the differentiated cell types. Sum of the relative signal intensities of all F ≥ 2 N-glycan signals in Fig. 2B was 25% for hESC, 13% for EB, and 11% for stage 3 differentiated cells, indicating that multifucosylation of sialylated N-glycans was gradually decreased during hESC differentiation. Structural analysis of these glycans is presented in the following section.

In contrast, a major group of N-glycan signals which increased during differentiation contained equal amounts of N-acetylhexosamine and hexose residues (N = H) in their monosaccharide composition, e.g. S1H5N5F1 (Fig. 3B). This is consistent with N-glycan structures containing non-reducing terminal N-acetylhexosamine residues. EB and further differentiated cells showed increased amounts of N-glycans expressing such monosaccharide compositions (marked with gray circles under Fig. 2B). Sum of the relative signal intensities of all N = H N-glycan signals in Fig. 2B was 3% for hESC, 14% for EB, and 22% for stage 3 differentiated cells, indicating that this structural feature was drastically increased during hESC differentiation at the EB stage and then further increased in stage 3 differentiated cells. However, these glycan structures were not characterized further in the present study.

Of the three sample types, only hESC were grown in the presence of hEF feeder cells, while both EB and further differentiated cells were grown without hEF. Therefore the observed hESC-specific glycans could have been contaminants derived from hEF. Figure 3 clearly shows that this was not true for the major hESC-associated glycan signal S1H5N4F2 (Fig. 3A) that had very low abundance in both analysed hEF samples. Similar examination of other hESC-associated glycans was consistent with this and we could conclude that any potential hEF-derived contamination was too minor to be detected. The major glycan structures that we identified to be associated with either the stem cells or the differentiated cells were not detected in the potential contamination sources. These structures included the major N-glycan signals with monosaccharide compositions indicating complex fucosylation and terminal HexNAc, for example the glycan signals shown in Fig. 3A–B. These signals were also undetectable in the cell culture media and supplements (data not shown).

The N-glycan profiles of the differentiated cells were also quantitatively different from the undifferentiated hESC profiles. A practical way of quantifying the differences between glycan profiles was to calculate the sum of the signal intensity differences between two samples. According to this method, the EB neutral and sialylated N-glycan profiles had undergone a quantitative change of 14% and 29% from the hESC profiles, respectively. Similarly, the stage 3 differentiated cell neutral and sialylated N-glycan profiles had changed by 15% and 43%. Taking into account that the proportion of neutral to sialylated N-glycans in hESC was approximately 2:1, the total N-glycan profile change was approximately 1/4 during the transition from hESC to stage 3 differentiated cells. The present data thus indicated that the mass spectrometric profile of the hESC N-glycome consisted of two discrete parts regarding propensity to change during hESC differentiation – a constant part and a changing part. As described above, even the minor signals reflected the differentiation stage associated N-glycan structural features. Therefore the mass spectrometric profile in itself was a specific and sensitive marker of hESC differentiation stage.

### Statistical analysis of N-glycan profiles

To evaluate statistical differences between hESC and differentiated cell N-glycan profiles, we performed one way ANOVA for each glycan signal (see Additional File 1). The analysis indicated statistical significance for the hESC-associated N-glycan signals, *e.g. *large high-mannose type N-glycan signals H7N2, H8N2, and H9N2, as well as complex fucosylated N-glycan signals H5N4F2, S1H5N4F2, and S1H5N4F3.

Factor and correlation analyses were employed in order to find common factors which could explain the observed distribution of N-glycan signals between the different cell types. The analyses were performed separately to the neutral N-glycans and acidic N-glycans (data not shown). Observation of the resulting factors indicated structure type related clustering of glycan signal groups in each factor. For example, major contributing signals in Factor N1 (explaining 26% of all variation in the neutral N-glycan fraction) reflected the balance between hESC-associated large high-mannose type N-glycans (H7N2, H8N2); and differentiation-associated low-mannose type N-glycans (H2N2, H3N2, H4N2), whereas Factor N2 (15%) was dominated by differentiated cell types associated glycan types: complex-type N-glycans with N = H type non-reducing terminal HexNAc (H5N5, H5N5F1) and hybrid-type N-glycans (H5N3, H5N3F1, H6N3). Correlation analysis indicated correlation among the large high-mannose type N-glycan signals (H6N2, H7N2, H8N2) and negative correlation with the small high-mannose type N-glycan H5N2.

In acidic glycan factor analysis, Factor A1 (explaining 24% of all variation in the acidic N-glycan fraction) reflected balance between 1) differentiated cell associated hybrid-type N-glycan signals (S1H5N3F1, S1H6N3) and 2) hESC associated complex-type N-glycans (S1H6N5F1, S1H7N6F1, S1H8N7F1) and complex-fucosylated glycans (S1H7N6F3). The major contributing signals in both Factors A2 and A3 (together explaining 23% of all variation in the acidic fraction) were N = H type terminal HexNAc bearing glycans (S1H5N5, S1H5N5F1, S2H5N5F1, S1H5N5F3, S1H6N6F1, S2H6N6F1).

In conclusion, the performed statistical analyses demonstrated that N-glycosylation changes during hESC differentiation were regulated so that the glycan biosynthetic changes were consistently similar from cell line to cell line, which was directly reflected in the level of cellular N-glycan profiles and even distinct glycan signals.

### Structural analyses of the major hESC N-glycans

**Figure 4:**
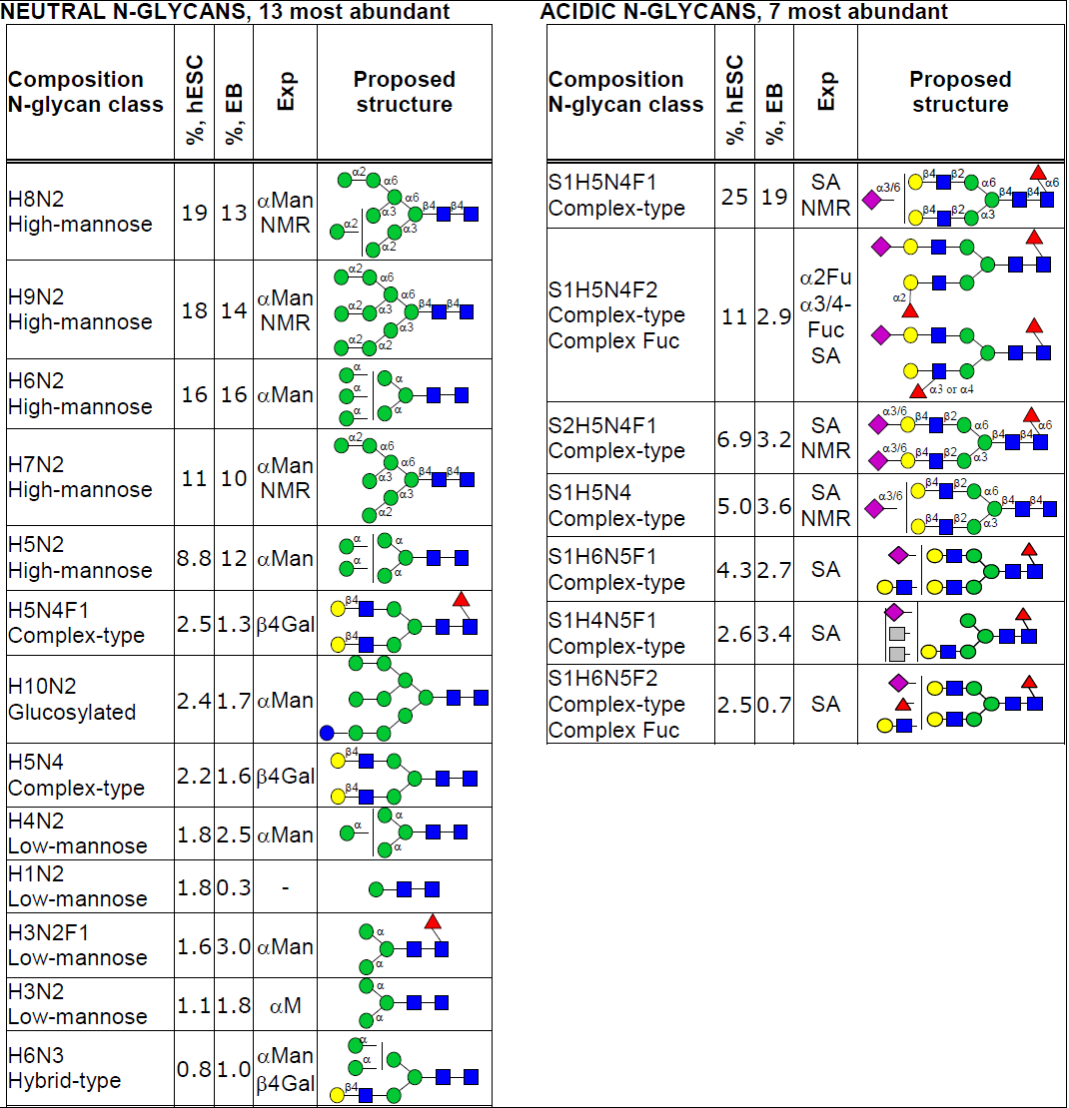
**Proposed structures for 20 most abundant N-glycan signals detected in the present study**. Proposed structures for 20 most abundant N-glycan signals detected in the present study (13 neutral and 7 sialylated N-glycans) are based on combined results from MALDI-TOF mass spectrometry, proton NMR spectroscopy (NMR), and exoglycosidase analyses with α-mannosidase (αMan), β1,4- and β1,3-galactosidase (β4Gal), β-N-acetylglucosaminidase, specific α1,3/4- and α1,2-fucosidases (α3/4Fuc and α2Fuc), and broad-range sialidase (SA). Relative abundances in hESC and EB N-glycan profiles are indicated. Only the positive identifications in ^1^H-NMR analyses and sensitivity to specific exoglycosidase digestions have been marked. Monosaccharide symbols are as in Figure 1. Where appropriate, glycosidic bonds have been indicated. Two simplifying assumptions have been made: i) in structures with H ≥ 3 and N ≥ 2, the proposed structures have been assigned a trimannosyl core structure, and ii) all fucosylated structures have been assigned a core fucose residue.

The N-glycan fractions were further analyzed by proton NMR spectroscopy recorded from N-glycans isolated from a larger sample of hESC grown on mEF. The assigned N-glycan structures are included in Figure 4 (for details see Additional File 1, Supplementary Fig. 3 and Supplementary Tables 1 and 2). In the obtained NMR spectrum of the hESC neutral N-glycans, signals consistent with large Man6-Man9 high-mannose type N-glycans were detected. No evidence of other structures was found since the high-mannose type N-glycan structures could explain all signals in the spectrum. In similar analysis of the sialylated N-glycan fraction, all the detected signals were consistent with biantennary complex-type N-glycans with type 2 N-acetyllactosamine (LacNAc) antennae, α2,6- and α2,3-linked sialic acids, and α1,6-linked N-glycan core fucose modifications. No signals corresponding to type 1 LacNAc antennae or other fucose linkages were detected. It was determined by integration of indicator signals that α2,6-linked sialic acids were more abundant than α2,3-linked sialic acids.

In order to validate glycan structure assignments made based on the mass spectrometric and NMR spectroscopic profiling analyses, we performed enzymatic degradation experiments with subsequent mass spectrometric detection (Fig. 4). Comparison of the original and digested N-glycan profiles allowed estimation of the relative proportions of non-reducing terminal monosaccharide residues of neutral N-glycans characteristic of hESCs (Fig. 4A). α-mannose residues were the most common terminal residues in the neutral glycan fraction, occurring in high-mannose type, low-mannose type, and hybrid-type N-glycan signals. β1,4-linked galactose residues occurred in hybrid-type and complex-type N-glycans, whereas terminal β1,3-linked galactose residues were not detected. Non-reducing terminal β-N-acetylglucosamine, α1,2-fucose, and α1,3- or α1,4-linked fucose residues were also present in minor glycan components (data not shown), but not in the major N-glycan signals presented in Figure 4.

The abundances of the major glycan signals in hESC and EB are indicated in Figure 4 and show distinct features in stem cells and differentiated cells. Most specifically, high-mannose type N-glycans were associated with hESC, while low-mannose type and hybrid-type N-glycans were more abundant in EB. These differences suggest that regulation of N-glycan biosynthetic pathways is changed during hESC differentiation. We are currently studying the potential regulatory mechanisms leading to these N-glycan profile differences (TJ *et al.*, manuscript in preparation). The observation of abundant low-mannose type N-glycans is interesting. We observed comparable amounts of fucosylated and non-fucosylated low-mannose type N-glycans in both hESCs and EBs, while analyses of the culture media revealed that they contained only non-fucosylated N-glycans. This indicates that these structures were produced by the studied cells and they did not originate from culture medium glycoproteins.

### Analysis of complex fucosylation in N-glycans of hESC

As noted above, there was a significant hESC association in N-glycans containing at least two deoxyhexose residues (F ≥ 2; see Fig. 2B). In contrast, glycan signals such as S2H5N4 that contained no deoxyhexose (F = 0) were increased in the differentiated cell types. This suggested that sialylated N-glycans in undifferentiated hESC were subject to more fucosylation than in the differentiated cell types. The most common fucosylation type in human N-glycans is α1,6-fucosylation of the N-glycan core structure [22] and this was also the major type of fucosylation detected in the present NMR profiling. In human N-glycans containing more than one fucose residue there should be other fucose linkages in addition to the α1,6-linkage [22,23], indicating complex fucosylation. The F ≥ 2 structural feature decreased as the cells differentiated, showing that complex fucosylation was characteristic of undifferentiated hESC.

**Figure 5:**
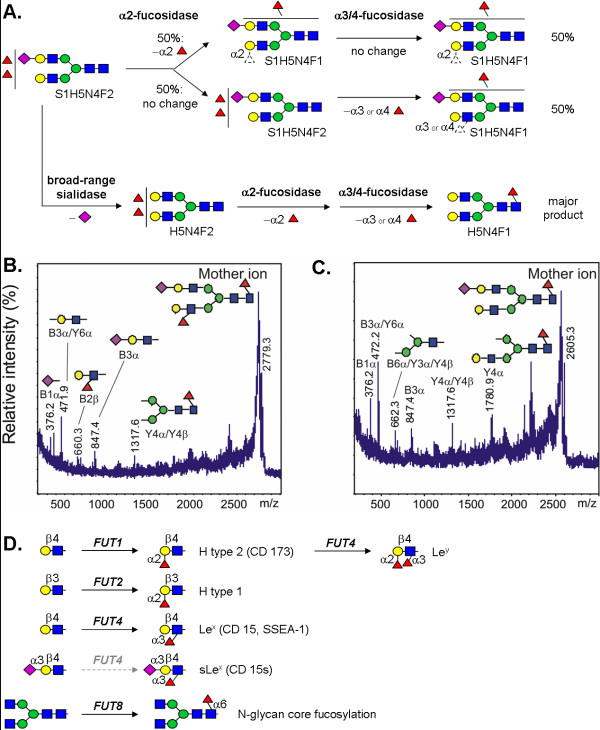
**Analysis of major complex fucosylated N-glycans of hESC**. **A**. The major sialylated N-glycan signal with complex fucosylation feature (S1H5N4F2) was subjected to exoglycosidase sequencing with linkage-specific fucosidases and broad-range sialidase. As outlined in the reaction scheme, the signal was shown to be an approximately 1:1 mixture of α1,2- and α1,3-/α1,4-fucosylated biantennary complex-type N-glycans with core fucosylation. **B. and C**. The acidic N-glycan fraction was permethylated and subjected to MS/MS fragmentation. Both S1H5N4F2 (**B**.) and S1H5N4F1 (**C**.) produced fragments that supported the structure assignments as indicated in the schematic drawings. **D**. The studied hESC lines were shown to express four of the known human fucosyltransferases. Minimal glycan acceptor and product specificities for the encoded enzymes are shown in the schematic drawing.

Exoglycosidase analysis of the sialylated N-glycan fractions was performed with specific α1,2- and α1,3/4-fucosidase enzymes to characterize the major hESC-specific N-glycan signals with complex fucosylation (Fig. 5A). Based on the sensitivity of the signal S1H5N4F2 towards the employed fucosidase enzymes, it was shown to include a mixture of isomeric N-glycan structures. Nearly half of the structures within S1H5N4F2 carried one terminal α1,2-linked fucose residue, while the other half carried one terminal α1,3- or α1,4-linked fucose residue. α1,3/4-fucosidase had a larger effect after desialylation (data not shown), indicating that in minor structures there was a fucose residue located subterminal to sialic acid. The majority of the structures contained exactly one fucose residue that was not susceptible to the employed fucosidase treatments, indicating fucosylation in the N-glycan core sequence. This was consistent with the NMR results showing that N-glycan core α1,6-fucosylation was the most abundant fucose linkage in hESC N-glycans. All detected desialylated and defucosylated N-glycans of hESC were sensitive to β1,4-galactosidase but not to β1,3-galactosidase digestion, indicating that the major N-glycan antennae were type 2 LacNAc. Taken together, the results suggested that the fucosylated antennae were either α1,2-fucosylated H type 2 *i.e. *Fucα1-2Galβ1-4GlcNAc or α1,3-fucosylated Le^x ^*i.e. *Galβ1-4(Fucα1–3)GlcNAc. Due to limited abundance of the minor structure with fucose residue subterminal to sialic acid, we were not able to sequence it, although a candidate structure is sLe^x ^*i.e. *Neu5Acα2-3Galβ1–4(Fucα1-3)GlcNAc.

MS/MS fragmentation analysis was applied to the most abundant hESC N-glycans with differential fucosylation, S1H5N4F2 (Fig. 5B) and S1H5N4F1 (Fig. 5C). The fragmentation patterns supported the exoglycosidase digestion and proton NMR profiling results and were consistent with sialylated biantennary complex-type N-glycans with predominant core fucosylation. Fucosylated antennae derived from S1H5N4F2 were represented by a single fragment ion at m/z 660.3 that fits to both H type 2 and Le^x ^terminal structures (Fig. 5B), while fragment ions corresponding to sLe^x ^were not detected.

Previously the gene expression profiles of the FES hESC lines have been determined [24] and we extracted the information of the expressed fucosyltransferases from this data (Fig. 5D). The hESCs expressed fucosyltransferase genes *FUT1*, *FUT2*, *FUT4*, and *FUT8*. Of these genes, *FUT1 *and *FUT4 *were overexpressed in hESC when compared to EB. As shown in Figure 5D, in N-glycans expressing type 2 LacNAc antennae (Galβ1-4GlcNAc) the functional expression of the corresponding glycosyltransferase enzymes may produce the following fucosylated epitopes: H type 2 (*FUT1*), Le^x ^and sLe^x ^(*FUT4*), Le^y ^(combined action of *FUT1 *and *FUT4*), as well as N-glycan core α1,6-fucosylation (*FUT8*). Structures corresponding to Le^y ^(difucosylated LacNAc) were not observed in the present study. Taken together, the evidence indicated that the major fucosylated epitopes in hESC N-glycans were H type 2 and Le^x^, and additional minor sialylated and fucosylated structures were most likely sLe^x^. However, based on the present experiments it could not be excluded that minor Le^y ^or type 1 structures could be present in hESC N-glycans.

### The identified hESC glycans can be targeted at the cell surface

**Figure 6:**
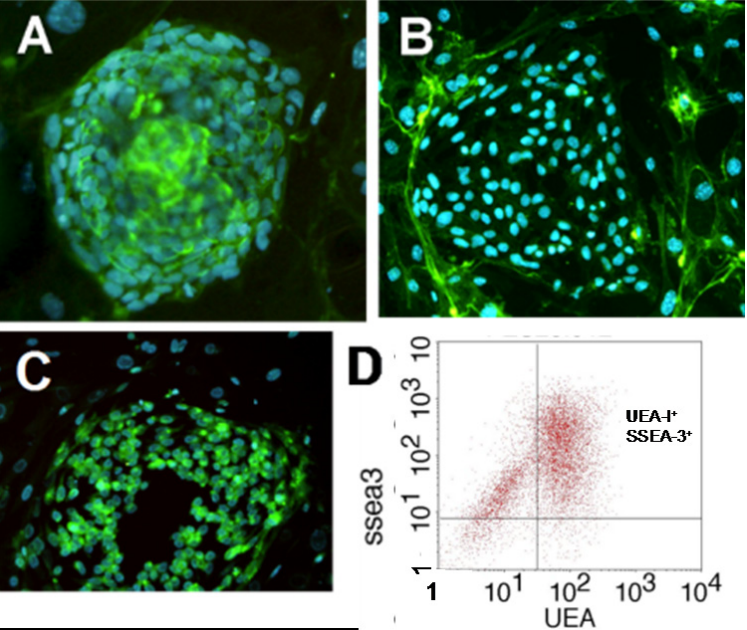
**Lectin staining of hESC colonies grown on mouse feeder cell layers**. **A**. *Maackia amurensis *agglutinin (MAA) that recognizes α2,3-sialylated glycans, preferably in type 2 LacNAc, stained hESC but not feeder cell surfaces. **B**. *Pisum sativum *agglutinin (PSA) that recognizes α-mannosylated glycans and core fucosylated N-glycans, stained only mouse feeder cell surfaces. **C**. *Ulex europaeus *agglutinin I (UEA-I) that recognizes α1,2-fucosylated glycans preferably within H type 2, stained the hESC colony. Mouse fibroblasts had complementary staining patterns with the lectins, indicating that their surface glycans are clearly different from hESC. **D**. Fluorescence-assisted cell sorting (FACS) diagrams of UEA-I selected hESC, showing that the majority of hESC were positive for cell surface UEA-I ligands.

From a practical perspective stem cell research would be best served by reagents that recognize cell-type specific target structures on cell surface. To investigate whether individual glycan structures we had identified would be accessible to reagents targeting them at the cell surface, we performed lectin labeling of three candidate structure types. Lectins are proteins that recognize glycans with specificity to certain glycan structures. Previous studies have extensively described lectin labeling of hESC [25-27] and we have also initiated a study of lectin and glycan antibody ligands on hESC surfaces (MM *et al.*, manuscript in preparation). In the present study, hESC colonies grown on mouse feeder cell layers were labeled in vitro by fluorescein-labelled lectins (Fig. 6). The hESC cell surfaces were clearly labeled by *Maackia amurensis *agglutinin (MAA) that recognizes structures containing α2,3-linked sialic acids, preferably α2,3-sialylated LacNAc, indicating that such sialylated glycans were abundant on the hESC cell surface (Fig. 6A). In contrast, the cell surfaces were not labelled by *Pisum sativum *agglutinin (PSA) that recognizes α-mannosylated glycans and potentially also core fucosylated N-glycans (Fig. 6B). However, PSA labelled the cells after permeabilization (data not shown). Finally, *Ulex europaeus *agglutinin I (UEA-I) that recognizes fucosylated structures, especially H type 2, stained hESC surfaces (Fig. 6C). The specificity of the lectin bindings was validated by inhibition with specific glycan inhibitors as described in the Methods section. Interestingly, the mouse fibroblast cells showed complementary staining patterns compared to hESC, suggesting that these lectin reagents efficiently discriminated between hESC and feeder cells. Figure 6D shows the FACS results with UEA-I further demonstrating that hESC were highly positive for complex fucosylated glycans including H type 2 terminal fucosylated sequences. Consistent with the lectin labeling results, the present structural analyses also demonstrated that both complex fucosylated structures including H type 2 and α2,3-sialylated LacNAc were abundant terminal structures in hESC N-glycans. The results further suggested that the identified glycan structures could be utilized to select reagents specifically targeting undifferentiated hESC, while not binding to other cell types. Glycans expressed on the hESC surface would be good targets for recognition by specific antibodies, which is subject of ongoing research in our laboratories.

## Discussion

In the present study, mass spectrometric and NMR spectroscopic analysis methods were applied in the first structural analysis of hESC N-glycan profiles. Previously, the glycosylation of hESC has been studied with lectins and antibodies [25-27], and a preliminary report has been published on mass spectrometric profiling of mouse embryonic stem cell (mESC) N-glycans [28]. The objective in the present study was to provide a global view on the N-glycan profile, or a "fingerprint" of hESC N-glycosylation, to structurally characterize the most abundant N-glycan structures of hESC, and to compare hESC N-glycosylation with differentiated cells. The hESC N-glycome was found to be characteristic to the cell type and different from either differentiated human cells or mESCs. The data provided information regarding the most characteristic features of hESC N-glycosylation and demonstrated that a dramatic N-glycan profile change takes place during hESC differentiation.

Over one hundred N-glycan signals were detected from each cell type. However, it is important to realize that many of the mass spectrometric signals in the present analyses include multiple isomeric structures and the one hundred most abundant signals therefore represent a larger amount of different glycans. The major N-glycans observed in hESC covered all the major N-glycan classes, namely oligomannose-type, hybrid-type, and complex-type N-glycans [29], and were decorated with sialylated and fucosylated antennae with equal complexity in all differentiation stages. This directly demonstrated that stem cell N-glycosylation was already as sophisticated as in the differentiated cells.

We found that the hESC N-glycan profile contained both a constant part and a variable part. The variable part was a sensitive indicator of the differentiation commitment. The major glycan types in the constant part were high-mannose type and biantennary complex-type N-glycans. The most characteristic feature of the variable part of the hESC N-glycome was complex fucosylation. In fact, it was found that 26% of the acidic N-glycan signals detected in hESC were multifucosylated. On the other hand, structurally different glycan structures were favoured by the differentiated cell types. About 1/4 of the total N-glycan profile of hESC changed during their differentiation. This demonstrated that during differentiation hESC substantially changed the appearance of their glycocalyx. New N-glycan features emerged in EB and further differentiated cells. These features included additional N-acetylhexosamine residues, potentially leading to completely new glycan epitopes presented on the differentiated cell surface. Such drastic changes in the N-glycome may profoundly alter both cell-cell interactions and the cells' responses to exogenous signals.

Glycans perform their functions in cells by acting as ligands for specific glycan receptors [30-32], functioning as structural elements of the cell [30], and modulating the activity of their carrier proteins and lipids [2]. More than half of all proteins in a human cell are glycosylated [3]. Consequently, a global change in protein-linked glycan biosynthesis can simultaneously modulate the properties of multiple proteins. It is likely that the large changes in N-glycans during hESC differentiation have major influences on a number of cellular signaling cascades and affect in profound fashion biological processes within the cells.

The major hESC-specific N-glycosylation feature we identified was complex fucosylation. Fucosylation is known to be important in cell adhesion and signalling events [22,23] as well as being essential for embryonic development [23]. Knock-out of the N-glycan core α1,6-fucosyltransferase gene *FUT8 *leads to postnatal lethality in mice [33], and mice completely deficient in fucosylated glycan biosynthesis do not survive past early embryonic development [34]. Fucosylated glycans such as the SSEA-1 antigen, a special form of Le^x ^[35-37], have previously been associated with both mESC and human embryonic carcinoma cells [15]. However, SSEA-1 is not expressed by hESC, which has previously been interpreted such that hESC would not express Le^x^. A recent report has suggested that mESC proliferation and differentiation can be influenced via specific recognition of fucosylated and sialylated glycoconjugates in a mESC line transfected with L1 receptor [56].

The published gene expression profiles for the same hESC lines as studied here [24] have demonstrated that four human fucosyltransferase genes, *FUT1*, *FUT2*, *FUT4*, and *FUT8 *are expressed in hESC (Fig. 5D), and that *FUT1 *and *FUT4 *are overexpressed in hESC when compared to EB (TJ *et al.*, manuscript in preparation). *FUT8 *encodes the N-glycan core α1,6-fucosyltransferase whose product was identified as the major fucosylated epitope in hESC N-glycans by the NMR analysis. The hESC-specific expression of *FUT1 *and *FUT4*, encoding for α1,2-fucosyltransferase and α1,3-fucosyltransferase enzymes, respectively [38], correlates with our findings of simple fucosylation in EB and complex fucosylation in hESC. The hESC-expressed enzyme product of *FUT2 *(Secretor) may primarily be linked to other glycan classes such as O-glycans or glycolipids based on its preference for type 1, 3, and 4 chains not detected in N-glycans (MM *et al.*, manuscript in preparation). Interestingly, the *FUT4*-encoded enzyme is capable of synthesizing both Le^x^, sLe^x^, and SSEA-1, although the capability to synthesize sLe^x ^may be low [39,40]. We detected N-glycan antenna structures consistent with Le^x ^in hESC N-glycans. Consistent with this, Le^x ^has been reported to be present in mESC N-glycans [28]. N-glycan signals potentially corresponding to sLe^x ^were detected in very low amounts in hESC, which is consistent with the reported specificity of the *FUT4*-encoded enzyme [39,40]. Our finding of H type 2 structures in hESC N-glycans is a novel feature that differentiates hESC from mESC. However, Wearne *et al. *[26] have already reported α1,2-fucosylation in hESC by utilizing UEA-I lectin staining. Significantly, product of the hESC-overexpressed fucosyltransferase *FUT1 *(H enzyme) is mainly responsible for H type 2 antigen biosynthesis (Fig. 5D). In conclusion, although hESC do not express the specific fucosylated antigen recognized by the SSEA-1 antibody, they share with mESC the characteristic features of complex fucosylation and expression of the Le^x ^antigen. The functions of these major fucosylation modifications in hESC remain to be elucidated in future studies. The present results suggest that the SSEA-1 antibody does not recognize Le^x ^when when presented on a biantennary N-glycan antenna.

Human embryonic stem cell lines have previously been demonstrated to have a common genetic stem cell signature that can be identified using gene expression profiling techniques [41-44]. Such signatures have been proposed to be useful in hESC characterization. In the present report we provide the first glycan profile signatures for hESC. The profile of the expressed N-glycans might be a useful tool for analyzing and classifying the differentiation stage in association with gene and protein expression analyses. In the present work we demonstrated that multiple mass spectrometric glycan signals correlated with the differentiation stage of hESC (Fig. 3). The present results suggest that N-glycan profiling could be developed into a tool for monitoring hESC differentiation status. Glycan profiling might be more sensitive than the use of any single cell surface marker and especially useful for the quality control of hESC-based cell products [21]. However, further analysis of hESC glycans may also lead to establishing new glycan structures as stem cell markers in addition to the commonly used SSEA and Tra glycan structures.

The present lectin staining experiments demonstrated that specific glycan molecules were abundant on the cell surface of hESC. The cell surface presentation of glycans makes them excellent targets for development of cell type specific recognition reagents. It seems plausible that knowledge of the changing surface glycan epitopes may be utilized as a basis in developing reagents and culture systems that would allow improved identification, selection, manipulation, and culture of hESC and their progeny. The present data allow rational selection and evaluation of glycan-specific antibodies based on knowledge of hESC glycan structures.

Venable *et al. *[25] and Wearne *et al. *[26,27] have extensively characterized hESC and EB reactivity for different lectins. Their results with MAA and UEA-I lectins confirm the present results about the expression of cell type-specific glycan epitopes on undifferentiated hESC surface. These previous studies may also provide cues for differentiation-associated N-glycan changes that were not structurally characterized in the present study. Venable *et al. *[25] described that N-glycans modified by bisecting GlcNAc residues as detected by *Phaseolus vulgaris *erythroagglutinin (PHA-E) were enriched in cells that had low or absent SSEA-4 staining, potentially indicating that such N-glycan structures were early signs of hESC differentiation. Wearne *et al. *[26] also found PHA-E ligands on differentiated hESC. It could be hypothesized that part of the terminal N-acetylhexosamine carrying N-glycans (e.g. N-glycans with N = H structural feature, see Fig. 2B) found in the present study to be associated with differentiation cells, could be modified by bisecting GlcNAc. Although specific N-glycan structural information is hard to extract from lectin binding profiles, the data of these earlier reports support our findings of abundant terminal α-mannose and LacNAc residues, both α2,3- and α2,6-sialylation, N-glycan core and peripheral fucosylation, as well as the presence of biantennary as well as branched complex-type N-glycans. However, specificities of individual plant lectins towards terminal mono-or oligosaccharide epitopes are usually not well characterized and may have multiple interpretations. Therefore the previous lectin studies gave a useful impression of the terminal monosaccharide epitopes but not exact structural information. The present structural data including larger oligosaccharide structures could have parallels with the prior data, but one should also consider technical factors which may explain differences or potentially cause artificial similarities. These differences include *e.g. *cell culture conditions, assay techniques, sample preparation, and differences between the studied cell lines. For example, we have previously shown that minor cell culture reagents may cause glycan contamination of stem cells [21].

By employing rapid purification and direct analysis of non-derivatized glycans we demonstrated mass spectrometric N-glycan profiling of the scarce hESC samples, enabling analysis of samples as small as 100 000 cells. In many glycomic studies of mammalian cells and tissues the isolated glycans have been derivatized (*e.g. *permethylated) prior to mass spectrometric profiling [45-48] or chromatographic analysis [49]. However, we chose to directly analyze the picomolar quantities of unmodified glycans and high sensitivity was achieved while omitting the derivatization and the subsequent additional purification steps. This straightforward method could be widely applicable to analysis and monitoring of stem cell lines. We have recently applied the same method to human cord blood hematopoietic cells [18] as well as human mesenchymal stem cells [50].

Stem cell glycosylation has been reported to be sensitive to composition of the cell culture medium [20,21]. In the present study, we analyzed all biological material in contact with the cells to exclude potential contamination sources. Since no cell type in the present study had identical cell culture conditions, we could not exclude the possibility that the observed profile differences were in part influenced by differences in cell culture. However, our data supports the conclusion that the major identified N-glycan structural features were not dependent on changes in either cell culture media or growth surface. hESCs and EBs were grown in the same culture medium except that bFGF was omitted from EB culture, while the major difference between hESC and EB culture was that hESC colonies were grown on feeder cells and EBs in suspension. To analyze if different growth surfaces could affect cellular glycosylation, we have compared N-glycan profiles of hESCs grown on hEF, mEF, Matrigel, and a defined non-animal growth support (MM *et al.*, manuscript in preparation). On all these surfaces, hESCs show an N-glycan profile typical to undifferentiated cells, including abundant complex fucosylation (such as S1H5N4F2, Fig. 3A) and low terminal HexNAc (such as S1H5N5F1, Fig. 3B). This suggests that the identified hESC-associated N-glycan profile features are not sensitive to changes in the growth surface. In addition, human fibroblast feeder cells, grown together with hESC, produce an N-glycan profile missing the key identifying characteristics of hESC glycosylation (for example complex fucosylation, Fig. 3A). Further, stage 3 differentiated cells grown as monolayers in different culture medium expressed the same differentiated cell associated structures as EBs (Fig. 1, 2, 3).

## Conclusion

Human embryonic stem cells have a characteristic N-glycan profile which undergoes major changes when the cells differentiate. Information regarding the specific glycan structures may be utilized in developing reagents for targeting these cells and their progeny. Future studies investigating the developmental and molecular regulatory processes resulting in the observed N-glycan profiles may provide significant insight into mechanisms of human development and regulation of glycosylation.

## Methods

### Human embryonic stem cell lines

Finnish hESC lines FES 21, FES 22, FES 29, and FES 30 were cultured as described previously [43]. Briefly, two of the analysed cell lines were initially derived and cultured on mouse embryonic fibroblast (mEF) feeders, and two on human foreskin fibroblast (hEF) feeder cells. For the present studies all of the lines were transferred on hEF feeder cells and cultured in serum-free medium supplemented with Knockout serum replacement (Gibco). To induce the formation of embryoid bodies (EB) the hESC colonies were first allowed to grow for 10–14 days whereafter the colonies were cut in small pieces and transferred on non-adherent Petri dishes to form suspension cultures. The formed EB were cultured in suspension for the next 10 days in standard culture medium without bFGF. For further differentiation (into stage 3 differentiated cells, St.3) EB were transferred onto gelatin-coated culture dishes in media supplemented with insulin-transferrin-selenium and cultured for 10 days.

For N-glycan profiling, on average 100 000 cells were collected mechanically from culture on hEF feeder cell layers, washed five times with phosphate buffered saline, and stored frozen until the analysis. In fluorescence-assisted cell sorting (FACS) analyses 70–90% of cells from mechanically isolated hESC colonies were typically Tra 1–60 and Tra 1–81 positive (data not shown). The differentiation protocol favours the development of neuroepithelial cells while not directing the differentiation into distinct terminally differentiated cell types [51]. Stage 3 cultures consisted of a heterogeneous population of cells dominated by fibroblastoid and neuronal morphologies. For more detailed structural analyses by NMR spectroscopy and glycosidase digestions, up to 10 million hESC were grown on mEF layers.

### Glycan isolation

Asparagine-linked glycans were detached from cellular glycoproteins by *F. meningosepticum *peptide:N-glycosidase F digestion (Calbiochem) and purified as described previously [18]. Briefly, the detached glycans were purified by sequential precipitation/extraction and solid-phase extraction steps with miniaturized chromatography columns of C_18 _silica, strong cation-exchange resin, porous graphitized carbon, and for the sialylated glycans also microcrystalline cellulose.

### Mass spectrometry and data analysis

MALDI-TOF mass spectrometry was performed with a Bruker Ultraflex TOF/TOF instrument (Bruker Daltonics, Germany) essentially as described [50]. Relative signal intensities of neutral and sialylated glycan components were assigned based on their relative signal intensities in the mass spectra when analyzed separately as the neutral and sialylated N-glycan fractions [52-55]. We calculated relative intensities for all detected glycan signals using the Flexanalysis 3.0 software (Bruker Daltonics). The present glycan profiles were extracted from the resulting signal lists by removing the effect of isotopic pattern overlapping, multiple alkali metal adduct signals, products of elimination of water from the reducing oligosaccharides, and other interfering mass spectrometric signals not arising from the original glycans in the sample. The resulting glycan signals in the presented glycan profiles were normalized to 100% to allow comparison between samples. The relative amounts of each glycan signal are expressed as "% of total profile" similarly as previously reported [17,18,50]. The mass spectrometric fragmentation analysis of permethylated glycans was performed using the Bruker Ultraflex TOF/TOF instrument according to manufacturer's instructions.

### Glycosidase analysis

Aliquots from the N-glycan fractions were subjected to digestion with α-mannosidase from Jack beans (*C. ensiformis*; Sigma-Aldrich); β1,4-galactosidase, β-N-acetylglucosaminidase, and α2,3-sialidase from *S. pneumoniae*; broad-range sialidase from *A. ureafaciens*; and β1,3-galactosidase, α1,3/4-fucosidase, and α1,2-fucosidase from *X. manihotis *(all from Calbiochem). Reactions with approximately 1–10 pmol oligosaccharide aliquots were carried out by overnight digestion at +37°C in 10 μl of 50 mM sodium acetate buffer pH 5.5. The activities of the enzymes in each reaction were optimised such that they had the following substrate specificities in control reactions: α2,3-sialidase digested Neu5Acα2-3Galβ1-4GlcNAc (Neu5Acα2-3LacNAc) but not Neu5Acα2-6LacNAc; β-N-acetylglucosaminidase digested GlcNAcβ1-3LacNAc but not GalNAcβ1-4GlcNAc; LacNAc but neither Galβ1-3GlcNAc nor Galα1-3LacNAc were digested with β4Gal; β1,3-galactosidase digested Galβ1-3GlcNAc; α1,3/4-fucosidase digested Fucα1-3(Galβ1-4)GlcNAc (Le^x^) but not Fucα1-2Galβ1-3GlcNAc; α1,2-fucosidase digested Fucα1-2Galβ1-3GlcNAc but not Le^x^; and α-mannosidase digested the high-mannose type N-glycans in a standard human N-glycan mixture. Digested glycan fractions were purified for analysis by solid-phase extraction and analyzed by mass spectrometry as described above.

### NMR methods

The isolated glycans were purified for the analysis by gel filtration high-pressure liquid chromatography in a column of Superdex peptide HR 10/30 (Amersham), with water (neutral glycans) or 50 mM NH_4_HCO_3 _(sialylated glycans) as the eluant at a flow rate of 1 ml/min. The eluant was monitored at 214 nm. Oligosaccharide pools were quantified against external standards N-acetylglucosamine and N-acetylneuraminic acid. Prior to NMR analysis the purified glycome fractions were repeatedly dissolved in 99.996% deuterium oxide and dried to omit H_2_O and to exchange sample protons. The ^1^H NMR spectra at 800 MHz were recorded using a cryo-probe for enhanced sensitivity [18].

### Lectin binding

Fluorescein-labeled lectins used in lectin binding studies were from EY Laboratories. Specificity of binding was controlled by inhibition with 50 mM α3'-sialyllactose (Kyowa Hakko Kogyo, Japan), 100 mM α-D-mannose methyl glycoside (Sigma-Aldrich), and 100 mM L-fucose (Danisco Sweeteners, Finland) for *Maackia amurensis *agglutinin (MAA), *Pisum sativum *agglutinin (PSA), and *Ulex europaeus *agglutinin-I (UEA-I), respectively. Fluorescence-assisted cell sorting (FACS) analyses were performed essentially as described [18].

### Statistical analysis

Normalized mass spectrometric N-glycan profile data from hESC (n = 4), EB (n = 7), and stage 3 differentiated cells (n = 6) were imported to Statistica 7.0 software (StatSoft). If all or almost all data values were zero, the corresponding signal was removed from the data set. In one way ANOVA with Fisher LSD post hoc analysis and Factor analysis signals having all or most of the values zero in certain cell type were omitted. Whisker box plots with means and standard deviations were developed and screened to have an overall view of the data and to identify mass peaks with variation between different cell lines or differentiation stage. Factor analysis was performed by principal component extraction; factor loadings were Varimax normalized and signals having factor loadings >0.62 and factors explaining >5% of variance were included into the model. Pearson correlation analysis was performed and correlations more than 0.7 or less than -0.7 were considered significant.

## Abbreviations

Abbreviations are as follows: bFGF: basic fibroblast growth factor; EB: embryoid bodies; ER: endoplasmic reticulum; F: deoxyhexose; FACS: fluorescence-assisted cell sorting; Fuc: L-fucose; G: N-glycolylneuraminic acid; Gal: D-galactose; GalNAc: N-acetyl-D-galactosamine; GlcNAc: N-acetyl-D-glucosamine; H: hexose; hESC: human embryonic stem cells; hEF: human foreskin fibroblast; H type 2: Fucα1-2Galβ1-4GlcNAc; ITS: insulin-transferrin-selenium; LacNAc: N-acetyllactosamine; Le^x^: Lewis x, Galβ1-4(Fucα1-3)GlcNAc; Le^y^: Lewis y, Fucα1-2Galβ1-4(Fucα1-3)GlcNAc; MAA: *Maackia amurensis *agglutinin; MALDI-TOF: matrix-assisted laser desorption-ionization time-of-flight; mEF: mouse embryonic fibroblast; mESC: mouse embryonic stem cells; N: N-acetylhexosamine; N-glycan: asparagine-linked glycan; Neu5Ac: N-acetylneuraminic acid; NMR: nuclear magnetic resonance; PSA: *Pisum sativum *agglutinin; S: N-acetylneuraminic acid; sLe^x^: sialyl Lewis x, Neu5Acα2-3Galβ1-4(Fucα1-3)GlcNAc; St.3: further (stage 3) differentiated cells; UEA-I: *Ulex europaeus *agglutinin-I. The schematic representation of oligosaccharides is in accordance with the guidelines proposed by the Consortium for Functional Glycomics  and as described in the legend of Figure 1.

## Authors' contributions

TS and AH contributed equally to this work. AH performed N-glycosylation analyses and TS analyzed the data and drafted the manuscript. MM, CO, TT, and TO provided the hESC samples. AH, MB, JH, TS, and JS developed glycan analysis techniques for stem cells. TJ analyzed gene expression data. MM and MT performed lectin binding assays. OA and AO performed the NMR analysis. JN participated in writing the manuscript. JS and JL supervised the project and contributed equally to this work. All authors read and approved the final manuscript.

## Supplementary Material

Additional file 1 **Supplementary data**. Supplementary data including the following supplementary figures and tables: **Supplementary Figures 1 and 2**. Examples of glycan profiling method evaluation. **Supplementary Figure 3 and Supplementary Tables 1 and 2**. NMR analysis of neutral and sialylated N-glycans. Click here for file
