# Supplementary material for: The N-glycome of human embryonic stem cells
Source: BMC Cell Biol. 2009 Jun 2;10:42. doi: 10.1186/1471-2121-10-42 (PMC2696424; doi:10.1186/1471-2121-10-42)
Supplement: Additional file 1 — Supplementary data. Supplementary data including the following supplementary figures and tables: Supplementary Figures 1 and 2. Examples of glycan profiling method evaluation. Supplementary Figure 3 and Supplementary Tables 1 and 2. NMR analysis of neutral and sialylated N-glycans. [file 1471-2121-10-42-S1.pdf]

Contents:

Supplementary Figure 1. Method evaluation. 2
Supplementary Figure 2. Method evaluation. 3
Supplementary Figure 3. NMR analysis of neutral N-glycans. 4
Supplementary Table 1. NMR analysis of neutral N-glycans. 5
Supplementary Table 2. NMR analysis of sialylated N-glycans. 6
Supplementary Table 3. One-way ANOVA of neutral N-glycan signals. 7
Supplementary Table 4. One-way ANOVA of acidic N-glycan signals. 8

Supplementary Figure 1. Method evaluation. Applicability of the method for relative quantitation of glycans was evaluated by mixtures of purified N-glycans. **A**. Mixture of three glycans at m/z 933, 1257, and 1485 was analysed. They yielded similar signal intensities (relative intensity RI was between 1.00 and 1.07, comparison to m/z 933 signal). **B**. Another glycan mixture was prepared, with the molar amount of glycan at m/z 1257 reduced to 50% and the molar amount of glycan at m/z 1485 reduced to 10%. The relative signal intensities reflected the change in the composition of the glycan mixture. RI of glycan at m/z 1257 was reduced to 58% of original and RI of glycan at m/z 1485 was reduced to 8.4% of original (comparison to m/z 933 signal). **C**. Example of the whole m/z range covering standard glycan mixture between 609-3569 Da for quality control of neutral N-glycan profiling.

Similar results were routinely obtained for acidic glycans analyzed in the negative ion mode (data not shown).

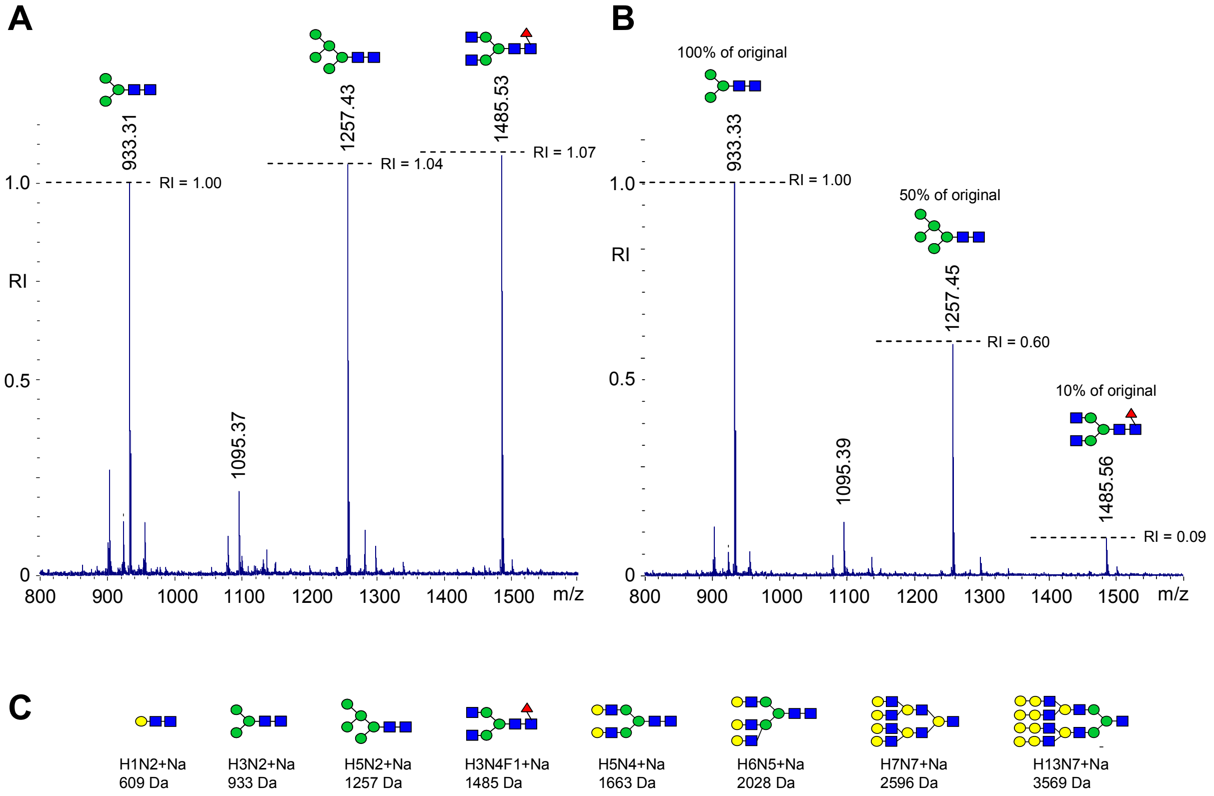

Supplementary Figure 2. Method evaluation. Neutral protein-linked glycan analysis performed by five different persons showing good reproducibility of the present method. m/z values refer to $[M+Na]^+$ ions.

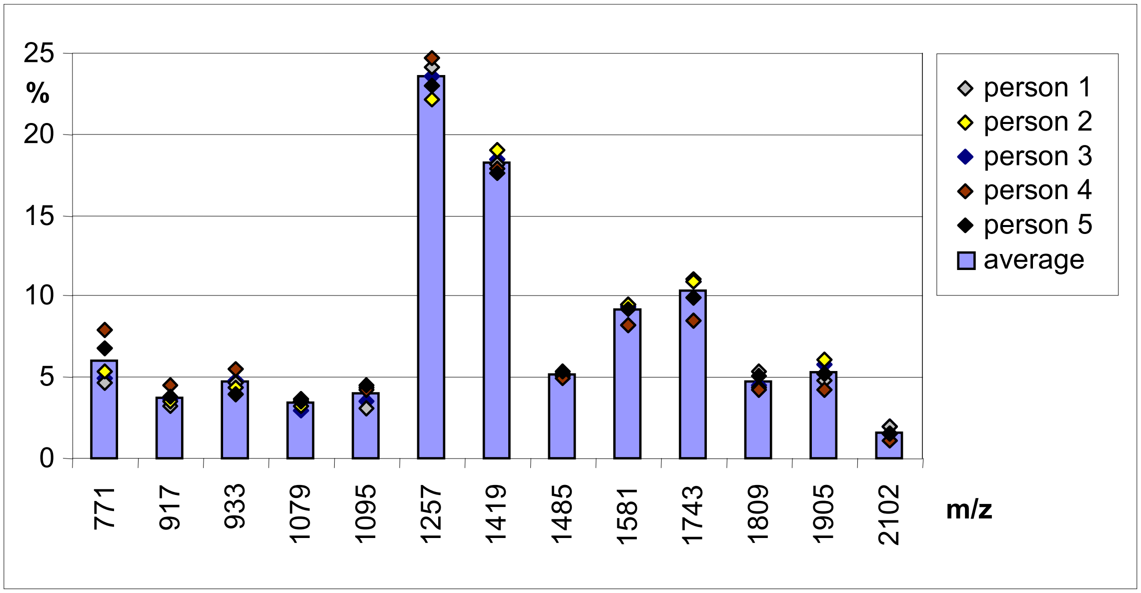

Supplementary Figure 3. ^{1}H-NMR analysis of the major hESC neutral N-glycans. The figure displays the anomeric proton region of the spectrum showing the structural reporter signals (Supplementary Table 2). The spectrum also included two major N-acetyl proton signals at 2.038 and 2.061 ppm (not shown). The chemical shifts are expressed in parts per million (ppm) by reference to internal standard acetone (2.225 ppm).

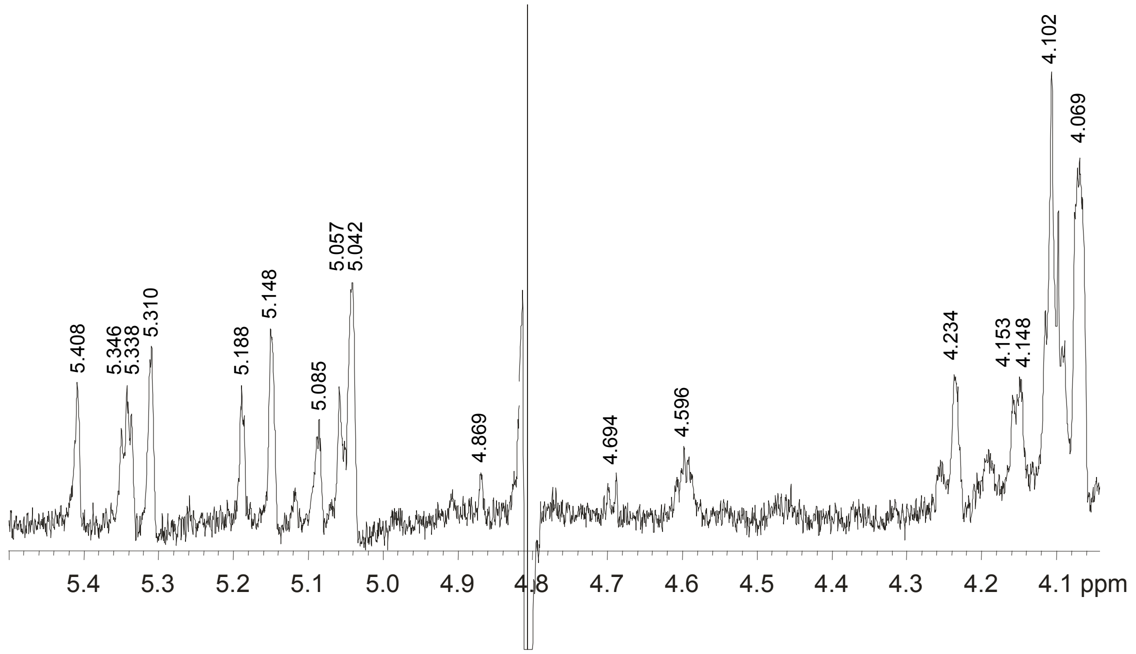

Supplementary Table 1. NMR analysis of the major neutral N-glycans of hESC. The identified signals were consistent with high-mannose type N-glycan structures such as the structures A-D that have monosaccharide compositions $H_{7-9}N_2$. The significant signals in the NMR spectrum can be explained by the following glycan structure combinations: A+B+C+D, A+B+D, A+C+D, B+C+D, A+D, or B+C. Reference data is after Fu *et al.* (Fu, D., *et al.*, 1994, Carbohydr. Res. *261*, 173-186) and Hård *et al.* (Hård, K., *et al.*, 1991, Glycoconj. J. *8*, 17-28). Monosaccharide symbols are as in the main text.

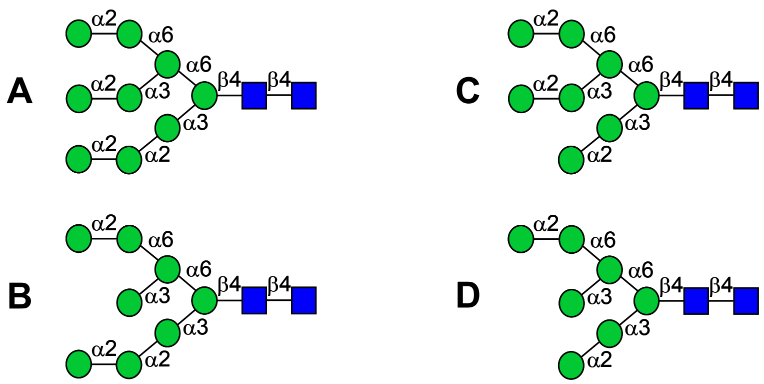

Glycan residue			^{1}H-NMR chemical shift (ppm)				
Residue	Linkage	Proton	**A**	**B**	**C**	**D**	**hESC** 1)
D-GlcNAc		H-1α	5.191	5.187	5.187	5.188	5.188
		H-1β	4.690	4.693	4.693	4.695	4.694
		NAc	2.042	2.037	2.037	2.038	2.038
β-D-GlcNAc	4	H-1	4.596	4.586	4.586	4.600	4.596
		NAc	2.072	2.063	2.063	2.064	2.061
β-D-Man	4,4	H-1	4.775	4.771	4.771	4.780	2)
		H-2	4.238	4.234	4.234	4.240	4.234
α-D-Man	6,4,4	H-1	4.869	4.870	4.870	4.870	4.869
		H-2	4.149	4.149	4.149	4.150	4.153
α-D-Man	6,6,4,4	H-1	5.153	5.151	5.151	5.143	5.148
		H-2	4.025	4.021	4.021	4.020	4.023
α-D-Man	2,6,6,4,4	H-1	5.047	5.042	5.042	5.041	5.042
		H-2	4.074	4.069	4.069	4.070	4.069
α-D-Man	3,6,4,4	H-1	5.414	5.085	5.415	5.092	5.408 / 5.085
		H-2	4.108	4.069	4.099	4.070	4.102 / 4.069
α-D-Man	2,3,6,4,4	H-1	5.047	-	5.042	-	5.042
		H-2	4.074	-	4.069	-	4.069
α-D-Man	3,4,4	H-1	5.343	5.341	5.341	5.345	5.346 / 5.338
		H-2	4.108	4.099	4.099	4.120	4.102
α-D-Man	2,3,4,4	H-1	5.317	5.309	5.050	5.055	5.310 / 5.057
		H-2	4.108	4.099	4.069	4.070	4.102 / 4.069
α-D-Man	2,2,3,4,4	H-1	5.047	5.042	-	-	5.042
		H-2	4.074	4.069	-	-	4.069

1) Chemical shifts determined from the center of the signal.
2) Signal under HDO.

Supplementary Table 2. NMR analysis of the major sialylated N-glycans of hESC. The identified signals were consistent with sialylated biantennary complex-type N-glycan structures such as the structures A-D that have monosaccharide compositions $S_{1-2}H_5N_4F_{0-1}$. Reference data is after Hård *et al.* (Hård, K., *et al.*, 1992, Eur. J. Biochem. *209*, 895-915) and Helin *et al.* (Helin, J., *et al.*, 1995, Carbohydr. Res. *266*, 191-209). The significant signals in the NMR spectrum can be explained by the structural components of these reference structures. Monosaccharide symbols are as in the main text.

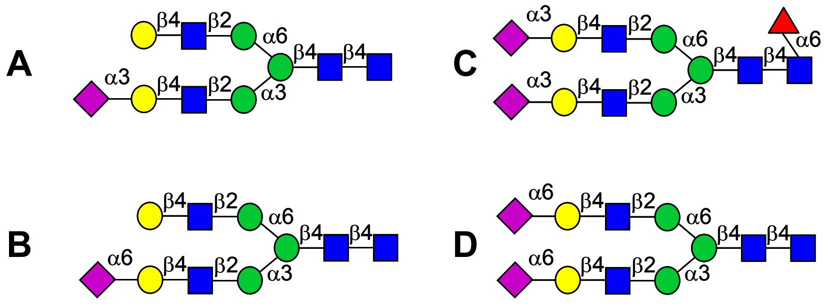

Glycan residue			^{1}H-NMR chemical shift (ppm)				
Residue	Linkage	Proton	**A**	**B**	**C**	**D**	**hESC** 1)
D-GlcNAc		H-1α	5.188	5.189	5.181	5.189	5.182 / 5.188
		NAc	2.038	2.038	2.039	2.038	2.038
α-L-Fuc	6	H-1α	-	-	4.892	-	4.893
		H-1β	-	-	4.900	-	4.893
		CH_3α	-	-	1.211	-	1.210
		CH_3β	-	-	1.223	-	1.219
β-D-GlcNAc	4	H-1β	4.604	4.606	n.a.	4.604	4.605
		NAc	2.081	2.081	2.096	2.084	2.081 / 2.095
β-D-Man	4,4	H-1	n.a.	n.a.	n.a.	n.a.	n.a.
		H-2	4.246	4.253	4.248	4.258	4.256
α-D-Man	6,4,4	H-1	4.928	4.930	4.922	4.948	4.927
		H-2	4.11	4.112	4.11	4.117	n.a.
β-D-GlcNAc	2,6,4,4	H-1	4.581	4.582	4.573	4.604	4.579 / 4.605
		NAc	2.047	2.047	2.043	2.066	2.047 / 2.069
β-D-Gal	4,2,6,4,4	H-1	4.473	4.473	4.550	4.447	4.447 / 4.472 / 4.545
		H-4	n.a.	n.a.	n.a.	n.a.	4.185
α-D-Man	3,4,4	H-1	5.118	5.135	5.116	5.133	5.118 / 5.134
		H-2	4.190	4.196	4.189	4.197	4.195
β-D-GlcNAc	2,3,4,4	H-1	4.573	4.606	4.573	4.604	4.579 / 4.605
		NAc	2.047	2.069	2.048	2.070	2.047 / 2.069
β-D-Gal	4,2,3,4,4	H-1	4.545	4.445	4.544	4.443	4.445 / 4.545
		H-3	4.113	n.a.	4.113	n.a.	n.a.

1) Chemical shifts determined from the center of the signal.
n.a.: Not assigned.

Supplementary Table 3. One-way ANOVA of neutral N-glycan signals. "x" denotes p-value < 0.05 and "y" equals 0.051 < p-value < 0.099. Highlighted p-values depict statistically significant association of the corresponding signal with hESC. Due to low n number, p-values < 0.099 were considered to be significant.

m/z	hESC - EB	hESC – St3
609		
730		x
771	x	x
892	x	x
917	x	x
933	x	y
1031		
1054	x	x
1079	x	x
1095	x	
1120	y	x
1136		
1209		
1216	x	y
1241		
1257	y	
1282		y
1298		
1323		
1339		y
1378	x	
1393		
1403	y	
1419		x
1428		
1444		
1460		
1485		
1501		y
1540	x	
1555		
1565		y
1581	x	x
1590		
1606		
1622		
1647		
1663		
1688		x
1702	x	x
1704		
1717		
1743	x	x
1752		
1768		
1784		
1793		
1809	x	x
1825		x
1850		x
1866		
1905	x	
1955	x	x
1971		
1987		
1996	y	y
2012		
2028	x	x
2041	y	y
2067	x	x
2101		
2117		
2142		
2158	y	
2174	x	x
2229		
2304		x

Supplementary Table 4. One-way ANOVA of acidic N-glycan signals. “x” denotes p-value < 0.05 and “y” equals 0.051 < p-value < 0.099. Highlighted p-values depict statistically significant association of the corresponding signals with hESC. Due to low n number, p-values < 0.099 were considered to be significant.

m/z	hESC-EB	hESC-st3
1354		x
1362		
1403		
1475		x
1500		x
1516		
1541		x
1549		
1557		
1563		
1565		
1637		x
1678	x	x
1703	x	x
1709		
1711		
1717		
1719	x	y
1727		
1744		y
1760		
1768	y	
1791		x
1799		x
1840		
1849		
1856		
1865		x
1873	x	y
1889	y	
1906	x	x
1914	x	
1928		
1930	x	x
1946	y	x
1947	x	x
1971		
1972		
2002	x	y
2010		x
2011		
2018		
2035		x
2051		
2052		
2060		
2068		x
2074		
2076	y	x
2082		
2092	x	x
2117		
2133		x
2156		x
2157		
2164		
2174		
2178		
2214		
2219		
2221		
2222	x	x
2230	x	x
2237		x
2238		
2239	x	
2246		
2253		y
2254		
2263		y
2279	x	x
2280		x
2293		
2295		
2302		
2305		
2319		
2320		
2321		
2349		
2365		
2367	x	x
2368	x	x
2376		
2383		x
2384		y
2390	x	x
2400		
2406		
2408	x	x
2424		
2425		
2441	x	y
2447	y	y
2448		
2457		
2482		x
2483		
2512		
2513		
2521		
2522		
2528		
2529		
2544		
2570		
2571		y
2586		
2587	x	x
2603		
2644	x	x
2645		y
2660		
2683		y
2714		
2732	y	
2733		
2791		
2806		
2807	y	
2812		
2878		
2879		
2880		
2886		x
2936		
2952		
2953	x	x
3024		
3025		
3026		
3098		
3099	x	x
3104		
3105		
3170		
3171		
3172	x	x
3244		
3389		
3390		
3463	x	x
